# Endoscopic Third Ventriculostomy for Obstructive Hydrocephalus Secondary to Delayed Intracerebellar Hematoma

**DOI:** 10.7759/cureus.17302

**Published:** 2021-08-19

**Authors:** Alejandro Ceja Espinosa, Jose Alfonso Franco Jimenez, Paola Reyes Vazquez, Guillermo Axayacalt Gutierrez Aceves, Aurelio Ponce Ayala

**Affiliations:** 1 Department of Vascular Neurosurgery, Instituto Nacional de Neurologia y Neurocirugia, Mexico City, MEX; 2 Department of Pediatric Neurosurgery, Instituto Materno Infantil del Estado de Mexico, Hospital Para el Niño, Toluca de Lerdo, MEX; 3 Department of Neurological Surgery, Instituto Mexicano del Seguro Social, Monterrey, MEX; 4 Department of Radioneurosurgery, Instituto Nacional de Neurologia y Neurocirugia, Mexico City, MEX; 5 Department of Neurological Surgery, Hospital Juarez De Mexico, Mexico City, MEX

**Keywords:** post-traumatic hydrocephalus, cerebellar hematoma, endoscopic third ventriculostomy, posterior fossa hemorrhage, neuroendoscopy, management of obstructive hydrocephalus

## Abstract

Nowadays, endoscopic third ventriculostomy (ETV) in neurosurgery has yielded good clinical results in various conditions. Intraventricular endoscopic procedures can be performed in different pathologies and not only in non-communicating hydrocephalus. This is presented accordingly in this clinical case.

We present the case of a patient who suffered a blunt traumatic brain injury (TBI) in the occipital region. Upon his arrival at the medical facility, he displayed altered neurological status and showed symptoms of aggressiveness, slurred speech, and gait ataxia. Initial non-contrast brain computed tomography scan presented a cerebellar traumatic subacute hematoma and secondary hydrocephalus. Therefore, we performed an ETV.

In most reported cases of cerebellar contusions among patients with TBI, the treatment was suboccipital craniectomy, clot evacuation, and external ventricular drainage (EVD). Unlike this case, the determined procedure was minimally invasive through ETV for the resolution of hydrocephalus with good clinical outcomes in addition to low morbidity and mortality. This case shows in the setting of delayed intracerebellar traumatic hematoma with secondary hydrocephalus being the main cause of neurological deterioration, a minimally invasive treatment such as ETV is suitable.

## Introduction

During the last decade, minimally invasive surgery has gained wide acceptance and popularity due to its excellent outcome and clinical results. In neurosurgery, the endoscopic third ventriculostomy (ETV) is universally performed for the treatment of primary and secondary hydrocephalus and its indications are broader every day [1].

Traumatic cerebellar hematomas or hemorrhagic contusions are rare in comparison to nontraumatic causes and may present acutely or in a delayed fashion when they are referred to as delayed traumatic intracerebellar hematoma [1].

The principal indication for intraventricular endoscopy is non-communicating hydrocephalus such as aqueductal stenosis, pineal, tectal or posterior fossa tumors, or fourth ventricle outlet obstruction but now is performed in different pathologies such as communicating hydrocephalus, normal pressure hydrocephalus, posttraumatic and posthemorrhagic with promising results. In 2002, Roux et al. described the use of ETV in one case of traumatic cerebellar hematoma with secondary hydrocephalus [2].

Even though there are multiple reports and analyses of outcome in ETV, it has not been clearly established in the setting of delayed hydrocephalus secondary to cerebellar traumatic hematomas its usefulness and there are still controversies regarding what is the best surgical approach to this subgroup of patients [3].

## Case presentation

**Figure 1:**
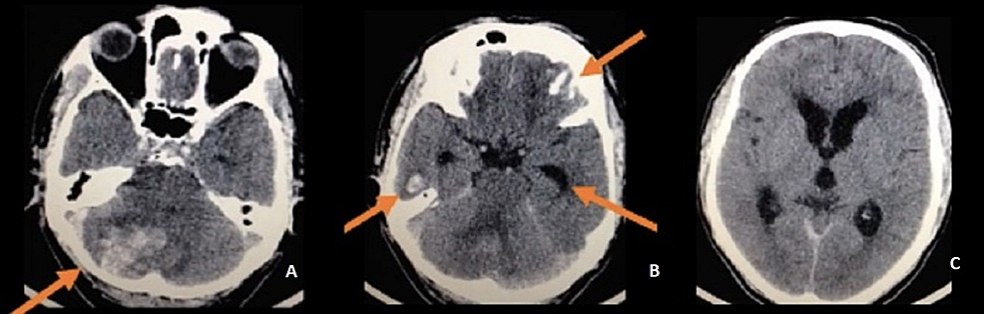
Initial non-contrast computed tomography (CT) scan (A) Axial view orange arrow showing cerebellar traumatic hematoma with a narrow fourth ventricle and lateral displacement. (B) Orange arrows showing wide temporal horns, temporal and frontal countercoup contusions. (C) Non-contrast CT scan with signs of hydrocephalus.

This case is about a 67-year-old man resident of Mexico State, who works as a construction builder. Past medical history is relevant for type II diabetes and hypertension both with adequate control. He arrived at our institution by ambulance to the emergency room due to altered mental status. Two days before admission, he suffered a fall from a ladder about 4 meters high that conditioned a blunt traumatic brain injury (TBI) to the occiput. Initial neurological status (referred by the patient wife) was 15 points in the Glasgow Coma Scale (GCS), adequate speech, cranial nerves without any deficit, and was able to walk, he did not present convulsions or sphincter alterations at the moment of trauma. Forty-eight hours after the initial trauma GCS was 12 points (E4 V3 M5) clinically he presented with aggressiveness, slurred speech, and gait ataxia. The initial non-contrast CT scan showed a cerebellar traumatic hematoma which was classified as Kirollos Grade 2, displacement of the fourth ventricle, right temporal posterior contusion, both temporal horns greater than 0.5 mm, rounded the third ventricle, transependymal migration in both frontal horns, left frontal contusion and traumatic subarachnoid hemorrhage in the right frontal and parietal lobes. The basal cisterns remained open (Figures 1A-1C).

**Figure 2:**
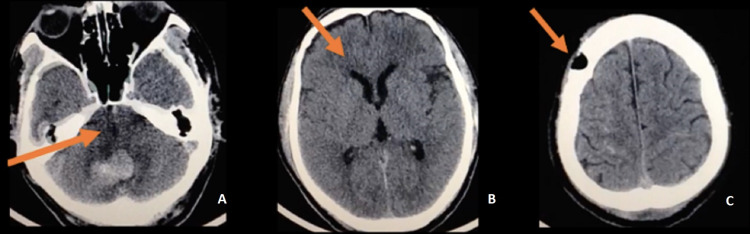
Postoperative non-contrast CT scan (A) Axial view orange arrow shows a central and open fourth ventricle. (B) Orange arrow shows the resolution of hydrocephalus. (C) Orange arrow shows the minimally invasive approach through Kocher's point.

With this clinical and radiological scenario, we concluded that the cause of the neurological deterioration was obstructive hydrocephalus and decided to perform an ETV. The surgery was done under general anesthesia, supine position with a soft headrest in the neutral position, and the selected approach was right precoronal (Kocher point) due to the less eloquence of the right cerebral hemisphere in comparison with the left side. We used a 30-degree rigid (Karl-Storz) endoscope to navigate within the lateral right ventricle then we entered the third ventricle through the right foramen of Monro, localize the mamillothalamic membrane and puncture it with a Fogarty #3 catheter, a 5-mm stoma was made to communicate the floor of the third ventricle with the basal cisterns. The operation was uneventful, no complications were reported and the patient was extubated right after the procedure GCS remained 12 points. The patient gradually improved, by 72 hours after surgery he regained consciousness to a GCS of 14 points, persisting only with some amnesia periods that were attributed to the frontal and temporal contusions, he was able to talk even though slurred speech persisted. He was discharged home four days after surgery. The postoperative CT scan showed the resolution of hydrocephalus (Figures 2A-2C).

At the follow-up three months after the surgery patient was with 15 points in the GCS, cranial nerves without any deficit, motor and sensory functions were normal, mild gait ataxia and slurred speech persisted. The three-month outcome was of 1 in the modified Rankin scale (mRs).

## Discussion

It is estimated that more than 1.7 million cases of traumatic brain injuries occur in the United States each year and nearly 3% of these cases result in fatality. Trauma to the posterior cranial fossa is a subset that affects the parts of the brain located inferior to the tentorium cerebelli. Intraparenchymal cerebellar hemorrhage secondary to trauma constitutes about 3% of all the traumatic head injuries and about 25% of all the traumatic injuries to the posterior fossa [4]. In comparison to post-traumatic intracerebral hematomas, traumatic cerebellar hematomas are rare and are reported in <1% of all head injuries. Various studies had reported the incidence of cerebellar hematoma from 0.4% to 3.7% of total intracranial hematoma [5].

In their clinical study on cerebellar contusion in 2015, Nashimoto et al. reported a frequency of 3.8% cerebellar contusions among patients with TBI, in all their surgical cases the treatment was conventional clot evacuation with suboccipital craniotomy and external ventricular drainage (EVD) [5].

The depressed level of consciousness in cases of cerebellar hematomas could be attributable to hydrocephalus, direct brainstem compression, and surrounding swelling, or both. In most cases, the decisions regarding surgical treatment are determined by the size of the hematoma and other factors, such as the presence of hydrocephalus, the degree of basal cisternal compression, and location of hematoma [6].

The last decade has seen an increased interest in the use of ETV. Consequently, the indications of this procedure have enormously increased, ranging from obstructive hydrocephalus to communicating hydrocephalus and biopsies. It has been published that the ETV is a suitable and successful option in cases of cerebellar non-traumatic hemorrhage and cerebellar infarct [7-9].

Hydrocephalus is caused by compression and occlusion of the fourth ventricle and the current surgical treatment is suboccipital craniectomy, clot evacuation, and EVD. This treatment presents various inherent risks to the prone position, prolonged intubation, and infection of the external drainage [10].

As reported by Kuramatsu et al., among patients with cerebellar hemorrhage, surgical hematoma evacuation is not associated with improved functional outcome, even though they conclude that investigation is necessary to establish whether there are different associations based on hematoma volume [11].

In 2019, Khattar et al. described the evacuation of cerebellar hematomas with a minimally invasive technique aided by the Artemis evacuation device with good results but it is no clear what was the cause of the hemorrhage in their patients we currently do not have access to this type of device in our institution, therefore, it was not considered a surgical option [12].

Even though we are aware of the risk that upward transtentorial herniation (UTH) is present in 2015 El-Gaidi et al., studied retrospectively 381 patients with obstructive hydrocephalus of which 87 were treated with ETV. Of these patients, 60 (69%) clinically improved, 26 (29.9%) remained stable, and only one (1.1%) suffered neurological deterioration and was diagnosed by a CT scan with a cerebellar hemorrhage, not UTH [13]. Also, in 2021, Moscardini-Martelli et al. reported that an ETV could be performed not even just in the setting of hydrocephalus but also as a treatment for UTH [14].

We decided to perform only the ETV given the subacute phase of the patient and because the clinical and radiological presentation was mainly due to hydrocephalus and intracranial hypertension not to the cerebellar contusion. The surgical course was uneventful and the patient had a good clinical outcome to mRs of 1, with this minimally invasive treatment.

## Conclusions

A minimally invasive ETV is a suitable option in cases of delayed intracerebellar traumatic hematoma with secondary hydrocephalus as this case shows. This indication of course must be individualized, and larger studies are needed to establish what is the best treatment for these patients.
